# The use of deep learning for smartphone-based human activity recognition

**DOI:** 10.3389/fpubh.2023.1086671

**Published:** 2023-02-28

**Authors:** Tristan Stampfler, Mohamed Elgendi, Richard Ribon Fletcher, Carlo Menon

**Affiliations:** ^1^Biomedical and Mobile Health Technology Lab, Department of Health Sciences and Technology, ETH Zurich, Zurich, Switzerland; ^2^Mobile Technology Group, Department of Mechanical Engineering, Massachusetts Institute of Technology (MIT), Cambridge, MA, United States; ^3^Department of Psychiatry, Massachusetts General Hospital, Boston, MA, United States

**Keywords:** digital health, deep learning, data science, public health, smartphone, activity recognition, physical activity, wearable technology

## Abstract

The emerging field of digital phenotyping leverages the numerous sensors embedded in a smartphone to better understand its user's current psychological state and behavior, enabling improved health support systems for patients. As part of this work, a common task is to use the smartphone accelerometer to automatically recognize or classify the behavior of the user, known as human activity recognition (HAR). In this article, we present a deep learning method using the Resnet architecture to implement HAR using the popular UniMiB-SHAR public dataset, containing 11,771 measurement segments from 30 users ranging in age between 18 and 60 years. We present a unified deep learning approach based on a Resnet architecture that consistently exceeds the state-of-the-art accuracy and F1-score across all classification tasks and evaluation methods mentioned in the literature. The most notable increase we disclose regards the leave-one-subject-out evaluation, known as the most rigorous evaluation method, where we push the state-of-the-art accuracy from 78.24 to 80.09% and the F1-score from 78.40 to 79.36%. For such results, we resorted to deep learning techniques, such as hyper-parameter tuning, label smoothing, and dropout, which helped regularize the Resnet training and reduced overfitting. We discuss how our approach could easily be adapted to perform HAR in real-time and discuss future research directions.

## Introduction

Human activity recognition (HAR) is an emerging field in health research that seeks to better understand human movements and behaviors (1). Researchers are increasingly working on HAR systems to translate measurements from wearable devices and smartphones into physical activity (2, 3). Analyzing data collected *via* the HAR systems remain challenging (4, 5). Moreover, understanding and modeling HAR data across different situations are needed for more personalized and effective interventions to improve health and wellbeing (6).

Digital phenotyping is defined as the moment-by-moment quantification of the individual-level human phenotype *in situ* using data from personal digital devices (7). Smartphones have become the device of choice for conducting digital phenotyping (6), as they have become a growing trend concerning the adoption rate over the last decade, even among senior adults (8). Beyond the pervasiveness of the smartphone, it has also improved many dimensions that make digital phenotyping with it increasingly convenient, notably its quantity and diversity of sensors, its sensor accuracy, its connectivity, its computing power, and its memory.

Studies have determined that users have their smartphones on them on average 36% of the daytime, a rate that increases when considering movement phases (9). Thus, the smartphone can easily transform into a convenient monitoring tool whose usage can be used in the form of apps covering several areas, such as mental health (9), regular health (10), or even fitness (11). The review by Straczkiewicz et al. (2) on HAR underlines the importance of finding a method that has the potential to generalize well, for example, from subjects in the laboratory to a free-living setting. It was also pointed out that HAR research should use make a more systemic use of publicly available dataset to benchmark their HAR recognition approach, a hint that we chose to follow for our study.

Some research also follows a multi-sensor approach for HAR, where the outputs of multiple sensors are conjointly analyzed to classify an activity (12). While this approach is likely to offer, in theory, a better performance perspective, it needs to be more practical. We will focus on a dataset employing the smartphone exclusively as a sensor.

In parallel, machine learning has greatly evolved during the last decade regarding performance and accessibility. Digital phenotyping revolves around types of tasks that machine learning is particularly well suited to tackle in a systematic way, such as regression or classification. In terms of time-series analysis, the focus of this article, deep learning architectures have replaced the paradigm of handcrafted feature engineering. Different families of neural net architectures such as CNN and RNN have been shown to outperform classical methods on benchmarks of more than 128 time-series datasets (13). As neural nets have evolved to deeper and more complex architectures, new architectures, such as Resnet, have emerged that incorporate features such as connecting non-contiguous activation layers, to address numerical problems such as vanishing or exploding gradient calculations. Therefore, we sought to optimize Resnet for HAR using data from a smartphone's accelerometer.

Developing robust models for HAR is challenging, and here, we attempt to optimize the development of these models. In this study, we will explore the use of deep learning for HAR and how it can be brought to improve state-of-the-art results. We will additionally discuss the challenges and limitations of our approach. The essential steps to conducting a deep learning-based HAR system will be addressed to pave the future development of this field.

## Method

We present below a description of the data and the deep learning architecture used for this research.

### Dataset choice

We chose to work with the UniMiB-SHAR dataset (14), whose data were collected in 2016. A prompt comparison with other popular datasets for human activity recognition from the literature is found in Table 1. Our main motivations for choosing the UniMiB-SHAR dataset are the following:

**Popularity**: It has over 300 citations, which indicates its intrinsic quality and provides a good opportunity to benchmark our results.
**Subjects**: It has a relatively high number of subjects and includes older people.
**Data quantity**: It contains 11,771 segments of both human activity and falls, covering nine classes of cleaned activity of daily living and eight classes of falls in total.
**Data quality**: It provides pre-processed data that are formatted, cleaned, and labeled.
**Availability**: It is publicly available and downloadable directly from the following website: http://www.sal.disco.unimib.it/technologies/unimib-shar/.

**Table 1:** Most popular datasets for human activity recognition and fall detection with accelerometer data.

**Dataset**	**Year**	Nr. Of citations^*^	**ADLs**	**Falls**	**Nr. Of Subjects**	**Age**
MobiFall (15)	2014	104	Yes	Yes	24	22–47
tFall (16)	2013	151	Yes	Yes	10	20–42
MobiAct (17)	2016	173	Yes	Yes	57	20–47
RealWorld (18)	2016	215	Yes	No	16	16–62
**UniMiB-SHAR** (14)	**2016**	**321**	**Yes**	**Yes**	**30**	**18–60**

We selected the most popular containing falls in terms of the number of citations. *According to Google Scholar search on 5 August 2022.

Bold values emphasize the dataset we picked for our study.

This dataset was built from scratch by researchers from the University of Milano Bicocca. They asked volunteers to place a smartphone in their pant pockets and to perform a series of activities and falls according to the protocol they established. Experiments were supervised by the researchers, and a queen-sized mattress was laid on the ground to prevent volunteers from injuring themselves by falling. Elders were excluded from falling activities due to safety concerns.

The recording device was a Samsung Galaxy Nexus I9250 with Android Version 5.1.1 equipped with a Bosh BMA220 acceleration sensor. The Android OS limits the acceleration range, and the sampling rate is capped at 50 Hz. The smartphone was alternatively placed in the front right and front left pant pockets.

### Data segmentation

Once time series were recorded, the researchers extracted the 3-s time windows from them to create segments for each activity or fall type. The rule followed was to search for a peak of 1.5 g in the data and to center a time window when one was found. If several consecutive values were above 1.5 g, the time window was centered on the first one. Notably, such preprocessing has one consequence to consider for the rest of the study: data leakage. Indeed, different segments can partially overlap and share common timestamps, which can lead to data leakage between the training and test sets. This point has been overlooked by all studies that have not performed leave-one-subject-out to assess the results.

**Figure 1:**
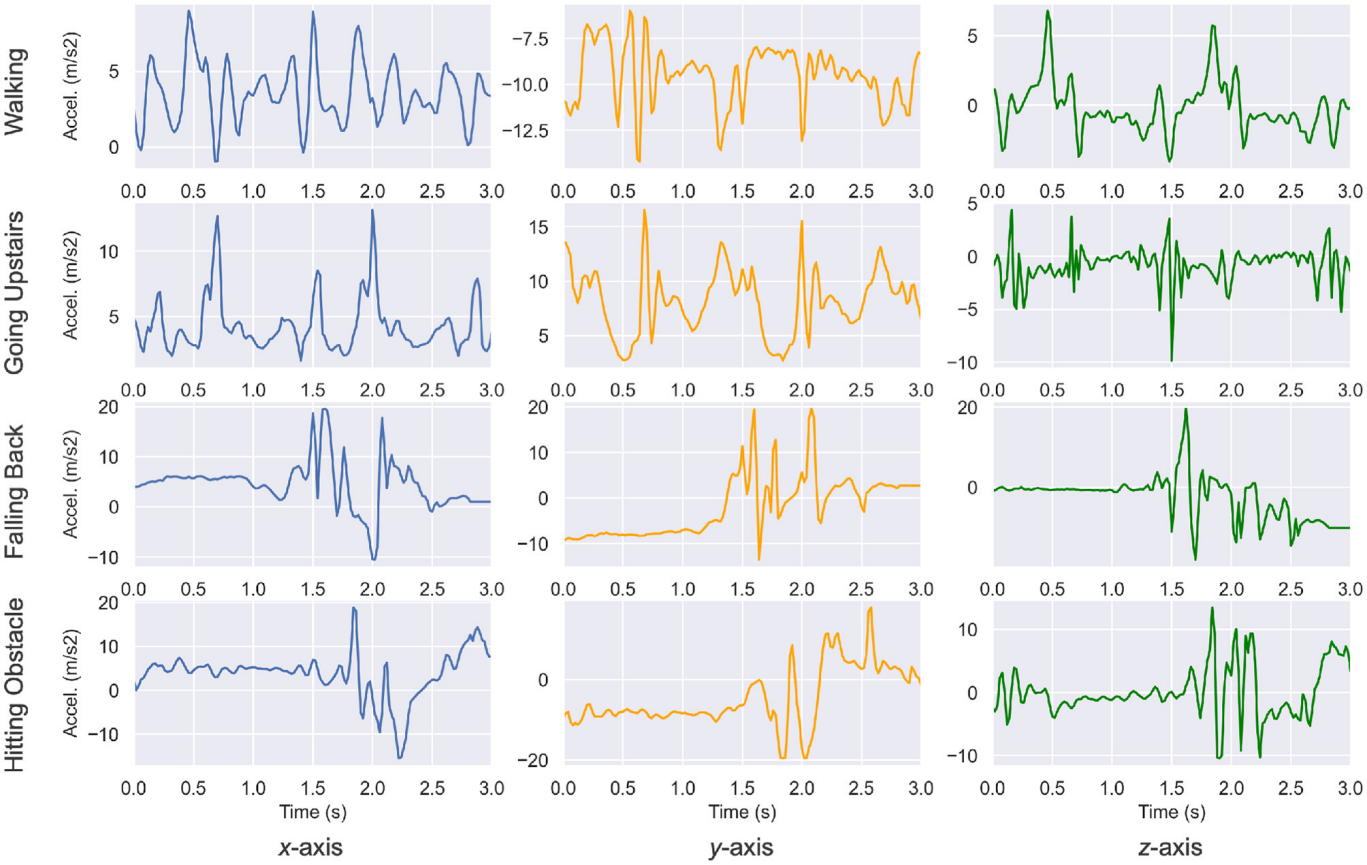
Acceleration segments from the UniMiB-SHAR dataset (14). Patterns and amplitudes variations are significantly different across various activities. For example, the walking activity features low amplitude and periodic patterns, whereas the falling back activity features a sudden burst of acceleration.

We first introduced the data from the UniMiB-SHAR dataset in Figure 1 with a plot of a few segments from the dataset. The acceleration value was plotted against the time (total duration of 3 s) and varied based on the movements of the phone carrier. The segments are centered on a maximum acceleration. One can already notice significant differences in patterns between ADLs and falls.

**Figure 2:**
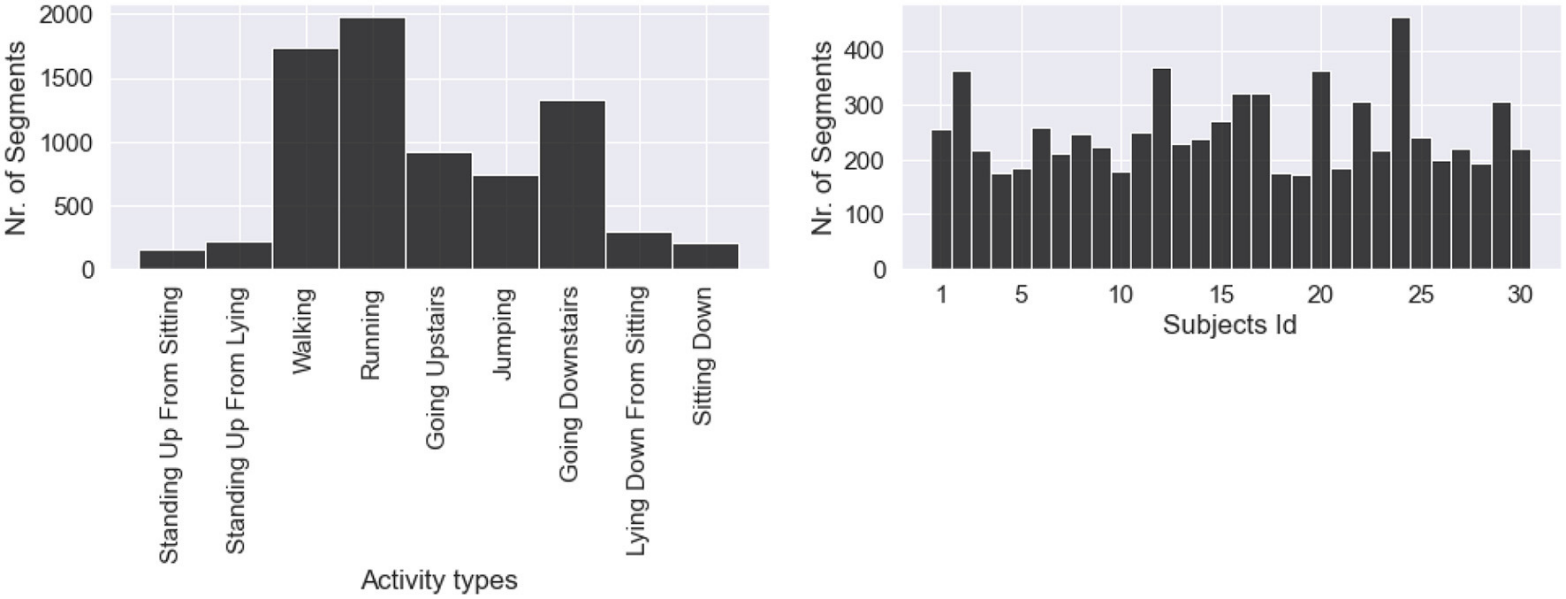
Distribution of daily living activities, with a strong class imbalance. Daily activities that are less cumbersome to perform are predominant (walking, running, going up, and downstairs).

**Figure 3:**
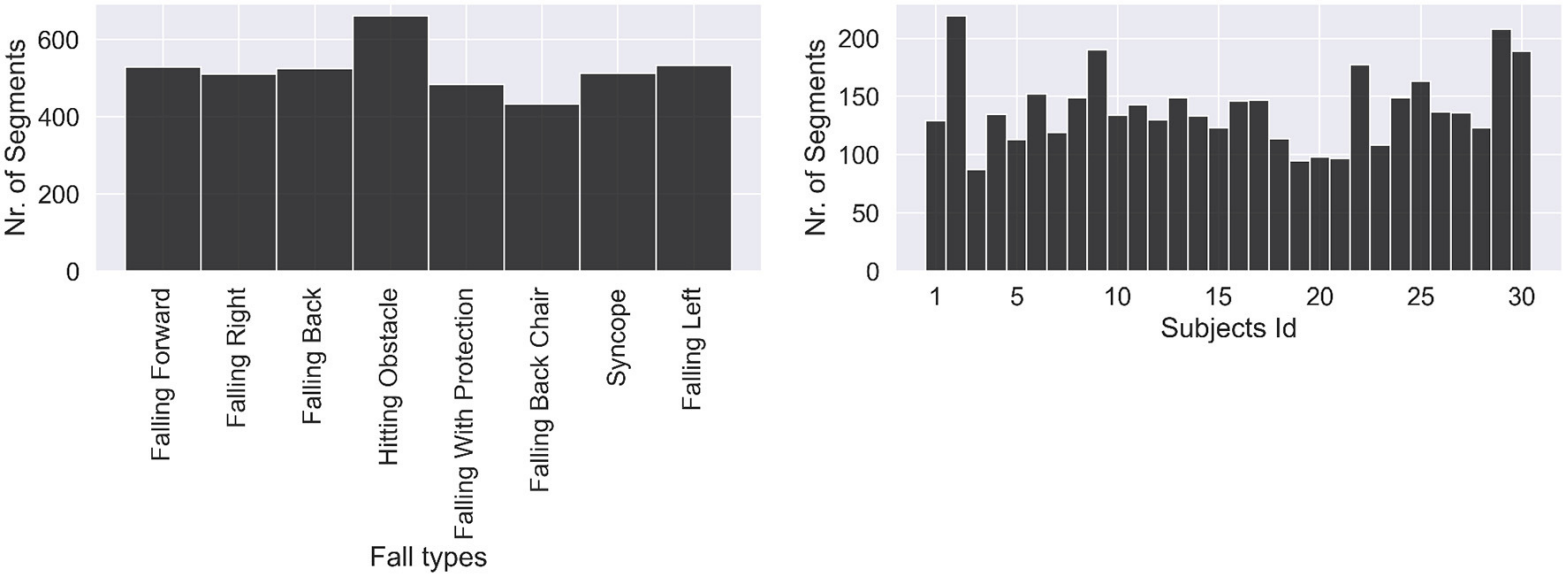
Distribution of fall activities. Classes are fairly balanced as all falls are about equivalent to perform.

**Figure 4:**
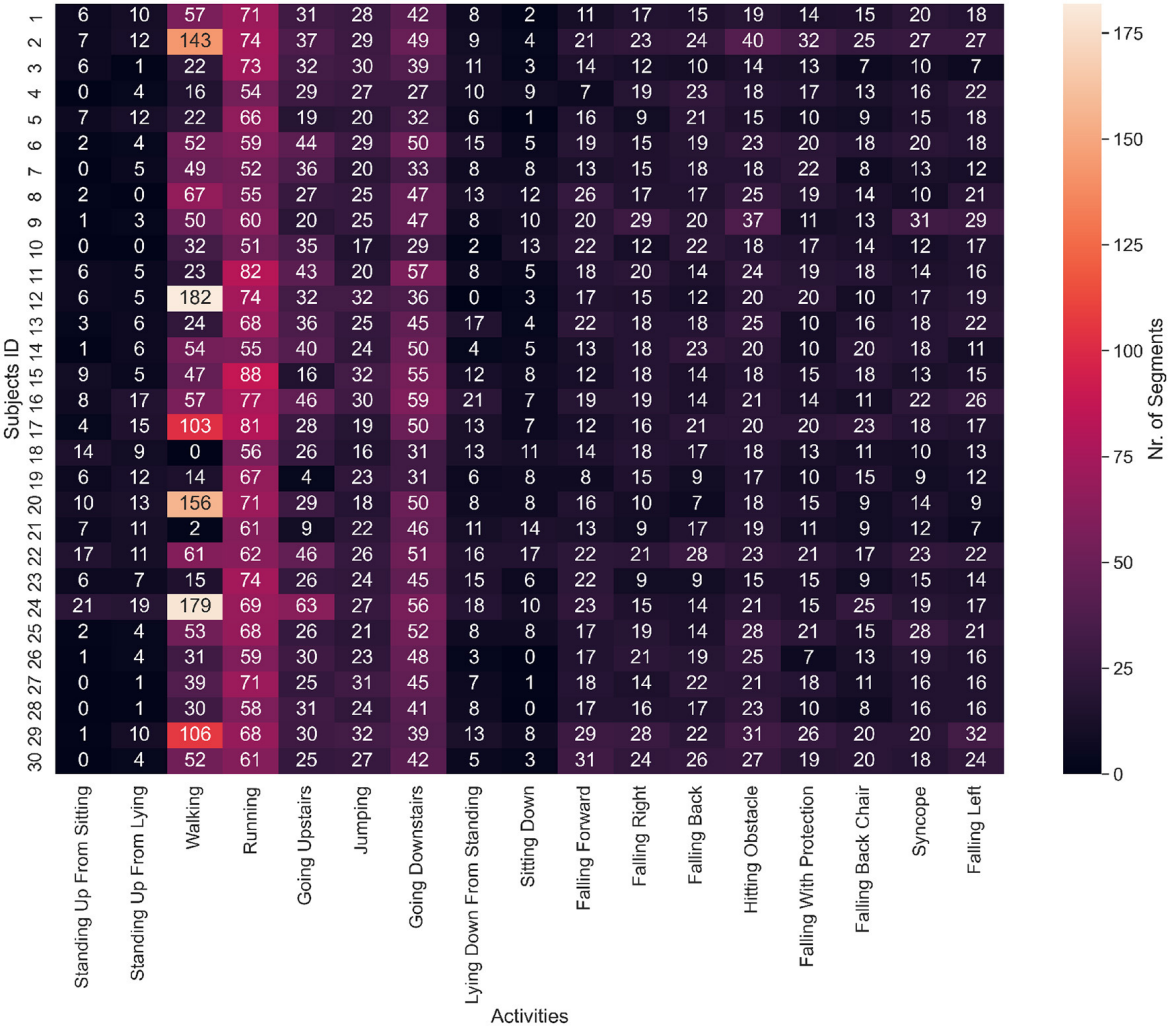
Heat map of the number of segments for each ADL or fall and subject. Subjects 4, 7, 8, 10, 12, 18, 26, 27, 28, and 30 are missing at least one type of ADL or fall. Walking, running and going downstairs are clearly the predominant activities by the number of segments.

Subsequently, we counted the number of segments for the nine classes of activities of daily living (ADLs) and eight classes of falls on two separate plots. Figure 2 shows how many segments from each ADL were in the dataset and how many ADL segments corresponded to each subject. We noticed a strong class imbalance. Cumbersome activities have fewer segments than the easiest ones, such as walking or running. Such imbalance will be addressed later by our choice of evaluation method. Figure 3 depicts how many segments from each fall type are in the dataset and how many fall segments correspond to each subject. There is only a minor class imbalance in the case of falls here. To cross-check whether each subject performed enough of each activity, we can dive one level of granularity deeper and plot the count of each ADL and fall per subject. Figure 4 demonstrates this distribution using a heat map. Beyond some strong class imbalances, we also notice that subjects 4, 7, 8, 10, 12, 18, 26, 27, 28, and 30 are missing at least one type of ADL or fall. We again noticed that walking and running were the activities with the most segments in the dataset.

### Statistical analysis

**Figure 5:**
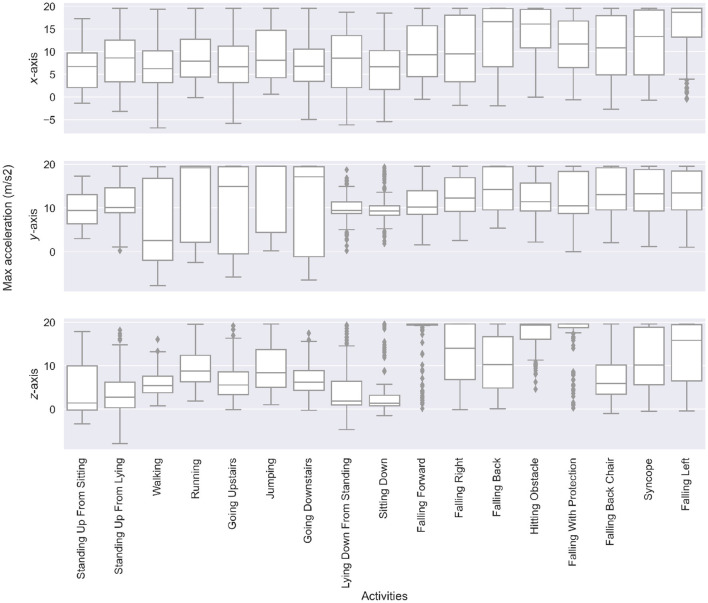
Boxplots of the maximum acceleration value along the *x*-, *y*-, and *z*-axis for each type of ADL and falls. Falls have significantly higher maximum accelerations than ADLs. Moreover, the direction of the fall is reflected in the differences of *x*-, *y*-, and *z*-axis accelerations.

Since segments are in the form of time-series data, we can conduct a statistical analysis to better grasp the differences between the ADLs and the fall groups. The core insight is that the maximum acceleration (i.e., the maximum magnitude value that can be found in a 3-s segment) tends to be higher for falls, especially on the *x*-axis and *z*-axis. We illustrate this with a boxplot of these maximum values for the whole dataset in Figure 5. Notably, because of the Android recording limitations, acceleration recordings are capped at 2 g, while some values could have been higher. The means of the three maximum distributions are rigorously compared with a *t*-test (19) in Table 2, which confirms falls that have higher accelerations.

**Table 2:** *t*-test of the means of maximum acceleration values for ADLs and falls, split by axis.

**Accelerometer axis**	**ADL mean (*n* = 7,579)**	**Fall mean (*n* = 4,192)**	***T*-test**	***p*-value**
*x*	7.72	12.31	–40.69	< 1e-6
*y*	10.10	12.89	–18.65	< 1e-6
*z*	7.22	13.56	–61.04	< 1e-6

The two groups are significantly different.

Leveraging these distinctions is not sufficient to build a classification algorithm. Some ADL segments may still have high maximum acceleration values; similarly, some fall segments may still have low maximum acceleration values. This is enough to rule out a classification approach entirely based on thresholds despite such an approach being present in the literature, as seen in the literature review.

### Deep learning Architecture

**Figure 6:**
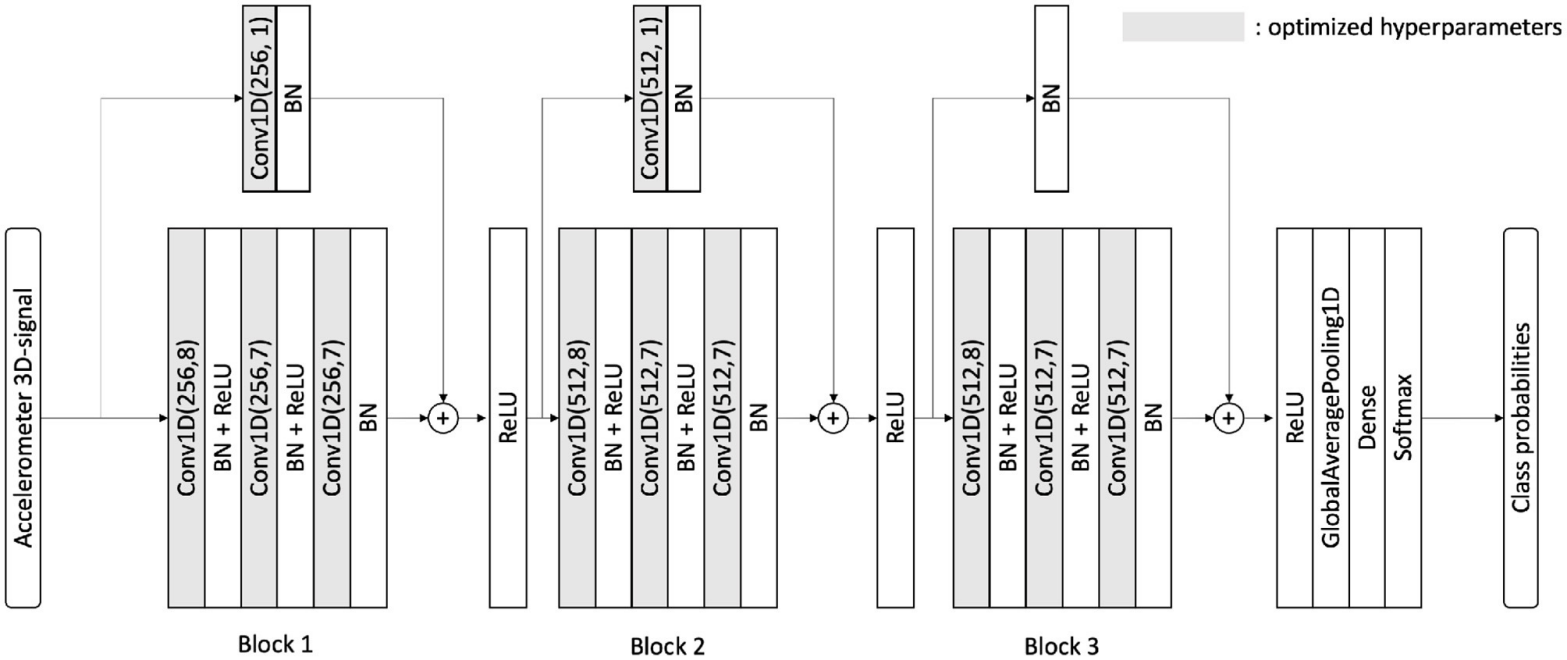
Neural architecture for our optimized Resnet. This is similar to both binary and multi-class classifications. The number of filters and the kernel sizes are different from the original architecture from Ismail Fawaz et al. (13). BN, batch-normalization; Conv1D, convolution 1D; ReLU, rectified linear unit; Conv1D(F, K), conv 1D with F filters and kernel size K.

We leverage deep learning to tackle this complex classification task. Deep neural networks have demonstrated remarkable performance across various datasets, such as image datasets. One family of deep networks, known as Resnets (20), often achieves state-of-the-art results on benchmark datasets. They enable better backpropagation in deeper architectures using skip connection over Resnet blocks. Ismail Fawaz et al. (13) have investigated a wide range of deep learning architectures for time-series classification, and they have proven that a Resnet would deliver the best results in most cases. After using a CNN, a Resnet, an encoder, and hybrid architectures, we chose a Resnet as our classifier for fall detection (binary classification) or human activity recognition (17 classes classification). The network architecture is shown in Figure 6, and it was originally published by Ismail Fawaz et al. (13). It differs from previously published Resnet architectures (21–23) benchmarked on the UniMiB-SHAR dataset in two main points: (i) Skip connections implement Conv1D operations (instead of a simple addition) to expand the number of channels and (ii) the kernels analyze more timestamps at once as we made them bigger (we use kernel of sizes 8 and 7 instead of the usual sizes 5 and 3). We call our approach optimized Resnet due to these changes and the further optimizations we introduced, which are detailed in the Discussion Section.

### Training and evaluation procedure

We leverage cloud computing on the Google Cloud platform to train our models. We created a notebook on the AI platform, running on the following software and hardware environment:

- Tensorflow Environment: Tensorflow 2.8
- Machine Type: 8 vCPUs, 30 GB RAM
- GPU: One NVIDIA Tesla T4

Several factors justify the need for cloud computing on a GPU for this task. From a deep learning perspective, the depth of the network, the use of dropouts, the number of segments (11,771), and the number of epochs (120) lead to high training time. From an evaluation perspective, cross-validation, and especially leave-one-subject-out (LOO), is time expensive, as the network may need to be retrained up to 30 times. Overall, the training and evaluation for each method can range from an hour to 2 days in the LOO case.

## Results

We now expose evaluation methods used and results and show our approach exceeds the current state of the art.

### Evaluation of binary and multi-class classifications

#### Binary classification

The binary classification is straightforward to explain; the network attempts to classify segments into two categories: ADL (0) and falls (1), while disregarding the activity or fall type. Obtaining high accuracy here is less challenging than in multi-class classification because there are only two possible output classes. As is often the case in the literature (as seen in the literature review) regarding human activity recognition, rather high figures can be achieved for the metrics presented. Our results are presented against various possible evaluation metrics in Table 3. The metrics are defined below for binary classification:

**Table 3:** Benchmark for binary classification of ADLs and falls.

**Evaluation method**	**Method**	**Algorithm**	**Accuracy (%)**	**F1 (%)**	**Sensitivity**	**Specificity**
5-fold CV	Micuci et al. (14)	SVM	98.71	N/A	N/A	N/A
	Proposed method	MLP	99.31	99.31	98.90	99.54
	**Proposed method**	**Opt. Resnet**	**99.87**	**99.87**	**99.90**	**99.85**
LOO	**Proposed method**	**Opt. Resnet**	**98.48**	**98.48**	**97.70**	**98.84**

Our approach yielded higher accuracy than the original UniMiB-SHAR article.

CV, cross-validation; LOO, leave-one-out; SVM, support vector machine; MLP, multi-layer perceptron; Opt. Resnet, optimized Resnet.

Bold values emphasize report results from our method.

We write *m* the total number of segments. We use the following notations: True positive (TP): A fall is correctly classified as a fall. True negative (TN): A non-fall activity is correctly classified as a non-fall activity. False positive (FP): A non-fall activity is incorrectly classified as a fall. False negative (FN): A fall is incorrectly classified as a non-fall activity.


$$\begin{array}{l} \text{Accuracy}=\frac{\text{TP}+\text{TN}}{m}\;\text{F1-score}=\frac{\text{TP}}{\text{TP}+\frac{1}{2}\left(\text{FP}+\text{FN}\right)}\\ \text{Sensitivity}=\frac{\text{TP}}{\text{TP}+\text{FN}}\;\text{Specificity}=\frac{\text{TN}}{\text{TN}+\text{FP}} \end{array}$$

We benchmark our approach against the results from Micuci et al. in the original article with the UniMiB-SHAR dataset. For further reference, we share metrics obtained for leave-one-subject-out (LOO). The implementation difference between cross-validation and LOO is that, in cross-validation, segments are randomly assigned between the train and validation set whereas in the LOO case, we ensure additionally that the train and validation sets of subjects (each subject has several recorded segments) are rigorously disjoint. In short, in LOO, the validation set is exclusively made of segments whose subjects were not in the train set. Thus, the LOO evaluation setting better represents the generalization potential of the algorithm as it faces entirely new data from entirely new subjects in the validation set.

However, these results should still be taken with a grain of salt. Indeed, a specificity that is not 100% will lead to the detection of false positives. For instance, in a case in which someone never falls, one false alarm will be triggered every 100 analyses. This rate can be computed simply by rewriting the formula that gives the sensitivity in the case of only negative segments, whereas in this case, *m* = *TN*+*FP*:


$$\text{Sensitivity}=\frac{\text{TN}}{\text{TN}+\text{FP}}\Rightarrow \text{FP}=m\times \left(1-\text{Sensitivity}\right)$$

#### Multi-class classification

We move to the case where we do not have two but 17 classes to classify. There are nine types of activities of daily living and eight types of falls. Classifying that many classes is more challenging and introduces more discrepancies into the literature. Near-perfect results of over 99% are far more challenging to attain, especially with the most rigorous evaluation methods. We use the two most common metrics that can be found in the literature, that is, accuracy and the weighted F1-score, which are defined later. We use the following notation: correctly classified (CC): The segment has been classified in its correct class. weight of class *i* (wi): Proportion of segments labeled *i* in the dataset. F1-score of class *i* (F1-score_*i*_): F1-score considering class *i* as positive and the rest as negative.


$$\text{Accuracy}=\frac{\text{CC}}{m}\text{ Weighted F1-score}=\overset{17}{\underset{i=1}{\sum }}w_{i}\cdot {\text{F1-score}}_{i}$$

We benchmark our results to compare approaches in Table 4 for the UniMiB-SHAR dataset. We structure and regroup the results from the literature based on their evaluation methods and compare them with our results. We found our approach to be state of the art in all cases.

**Table 4:** Benchmark of multi-class classification on the UniMiB-SHAR dataset.

**Evaluation method**	**Details**	**Authors**	**Algorithm**	**Accuracy (%)**	**Weighted F1 (%)**
Validation set	30% validation	Tang et al. (21)	Triplet attention Resnet	N/A	78.55
		Tang et al. (24)	Lego CNN	72.80	74.46
		Gao et al. (25)	SK-CNN	76.84	N/A
		Teng et al. (23)	Local Resnet	80.90	80.66
		Al-qaness et al. (22)	Multi-ResAtt RNN	84.99	85.06
		Cheng et al. (26)	Conditional CNN	88.63	N/A
		**Proposed method**	**Opt. Resnet**	**96.18**	**96.17**
Test set	10% test	Tang et al. (27)	HS-CNN	79.02	79.19
		**Proposed method**	**Opt. Resnet**	**98.71**	**98.71**
	20% test	Xu et al. (28)	Deformable Resnet	80.02	N/A
		**Proposed method**	**Opt. Resnet**	**98.64**	**98.65**
CV	5-fold	Huang et al. (29)	Shallow CNN	75.42	N/A
		Teng et al. (30)	Layer-wise CNN	78.07	77.82
		Mekruksavanich et al. (31)	LSTM-XGB	92.59	N/A
		Lv et al. (32)	ConvLSTM	95.30	97.30
		**Proposed method**	**Opt. Resnet**	**97.39**	**97.45**
	10-fold	Vong et al. (33)	XGBoost	91.22	86.40
		**Proposed method**	**Opt. Resnet**	**98.07**	**97.98**
LOO	30-fold	Lv et al. (32)	ConvLSTM	N/A	78.40
		Li et al. (34)	Hybrid CNN-LSTM	77.03	75.93
		Jin et al. (35)	CNN-D	78.24	77.59
		**Proposed method**	**Opt. Resnet**	**80.09**	**79.36**

Our approach outperforms the state-of-the-art approach across all kinds of evaluation methods. CV, cross-validation; LOO, leave-one-out; CNN, convolutional neural network; SK, selective kernel; Multi-ResAtt, multi-level residual network with attention; RNN, recurrent neural network; Opt. Resnet, optimized Resnet; LSTM, long-short term memory; XGB, extreme gradient boosting; CNN-D, CNN with distance loss.

Bold values emphasize report results from our method.

## Discussion

We discuss here the key differences and improvements our approach brings compared with other approaches from our benchmark. We eventually underline some machine-learning-related limitations.

### Optimization and deployability of deep learning

We started from the base Resnet architecture of Ismail Fawaz et al. (13), which is available at the following GitHub repository: dl-4-tsc. We only considered the architecture and did not perform in any kind of transfer learning. Subsequently, we conducted a series of hyperparameter optimizations, which are summarized in Table 5. A simple ablation study to show the contributions of the different techniques used is given in Table 6, for the case of leave-one-subject-out as this is the most challenging case to regularize. For optimizations regarding the network, we mostly increased the number of parameters of the filters and the kernels from 581,698 parameters for the standard Resnet to 11,567,362 parameters for our optimized Resnet. This mostly made training and evaluation time rise (the 30-fold cross-validation took approximately 36 h to complete). However, despite 11 million parameters being seemingly daunting for deployment purposes, our model could still be successfully run live with no prediction delays when we deployed it on a Samsung Galaxy A22 5G smartphone running Android 11. For optimizations regarding training, we found increasing the number of epochs necessary due to the higher number of parameters, and we could partially compensate for the training time by increasing the batch size (i.e., the number of segments given simultaneously to the network during the training phase). Smoothing labels is a common trick to boost deep learning network performance. The idea is to change the one-hot vector class representation to a probabilistic representation where other classes have small yet non-zero probabilities. Consequently, the following has been demonstrated:

**Table 5:** Our hyperparameters tuning of the proposed optimized Resnet network.

**Category**	**Element**	**Description**
Network	Conv1D	Increased filter sizes from (64,128,128) to (256, 512, 512) for all Resnet blocks
	Conv1D	Increased kernel sizes from (8,5,3) to (8,7,7) in all Resnet blocks
Training	Labels	Smoothed labels with α = 0.1
	Batch size	Increased training batch size to 256
	Dropout	Introduced Dropout with rate = 0.5
	Epochs	Set training epochs to 120

We found these values to yield the best accuracy and F1-score.

**Table 6:** Simple ablation study on multi-class classification with LOO evaluation for the optimized Resnet.

**Evaluation method**	**Authors**	**Steps**	**Accuracy (%)**	**F1 (%)**
LOO	Ismail Fawaz et al. (13)	Resnet base architecture	77.89	76.85
	-	+ Increase kernel and filter sizes	79.02	78.17
	-	+ Label smoothing	79.33	78.54
	**Proposed method**	+ Dropout	**80.09**	**79.36**

Epochs are 120, and batch size is 256. The biggest improvement is brought by increasing the number of parameters (in kernels and filters) in the network.

Bold values emphasize report results from our method.

Label smoothing influences representations learned by the penultimate layer of the network and encourages representations of training examples from the same class for grouping in tight clusters (36). This results in the network's enhanced ability to distinguish classes. The principle for label smoothing is to change the encoding of classes from one-hot vectors to smooth vectors (36). Smoothing can be seen as a function *f*_α_, with smoothing parameter α, that for a one-hot vector *v*_*k*_: = (_δ_*i, k*_)*i*_ representing class *k* (among *K* possible classes) returns $\tilde{v_{k}}$, the α-smoothed vector. In our case, we found the case α = 0.1 to yield the best results.


$$f_{\alpha }:v_{k}=(0,...,0,1,0,...,0)\to {\tilde{v}}_{k}=(\frac{\alpha }{K},...,\frac{\alpha }{K},1-\alpha +\frac{\alpha }{K},\frac{\alpha }{K},...,\frac{\alpha }{K})$$

Dropout is an another common supervised deep learning trick that prevents the network from overfitting (37). Randomly dropping activation units during the training phase forces the network to not be overreliant on only a few of them and makes it more robust. We found it very useful in our case; accuracy on the training set was always near 100% at the end of the training but much lower on the validation set.

### Related work from benchmark

Our benchmark regroups head-to-head 17 articles (21–35) by sharing their approach concerning the human activity recognition task evaluated on the UniMiB-SHAR dataset. Deep learning was the method of choice in almost every case (21–26, 28–32, 34, 35) to try to achieve state-of-the-art results. Only Vong et al. (33) employed a more standard machine learning and feature engineering approach. When choosing deep learning, the focus of studies was split into two parts. First, the developed classification method should achieve the highest accuracy or F1-score known to the authors. Second, some authors mentioned that their deep learning architecture should be kept reasonable in terms of memory consumption and computation time (23, 24, 26, 29, 30). This is only logical from an operational perspective, as the final goal of the algorithm is to be embedded into a portable device and to run live, which some studies have tested with their solutions (21, 26, 28, 29).

The question of feature representation in the case of human activity recognition was also raised as is often the case in deep learning studies. Li et al. argued that their hybrid CNN-LSTM allowed the extraction of both long- and short-term dependencies in the data (34). Tang et al. and Al-qaness et al. both leveraged attention mechanisms in their network (21, 22), and Lv et al. designed a module to specifically maximize information through the network by adding connections (32). Kernels and filters are hyperparameters found in CNN or LSTM architectures and are particularly appropriate for analyzing time-series data. Filters were the focus of a dimensional optimization by Tang et al. (24) and Gao et al. (25) introduced a selective kernel mechanism for convolutions.

Beyond architecture and operations, training loss was researched, and a clear trend toward building local losses and layer-wise training appeared in three selected studies (23, 24, 30). The creation of a clustering loss, the class anchor clustering (CAC) loss, was published in Jin et al. (35)

Finally, despite the UniMiB-SHAR dataset being seen as a benchmark dataset by all of the aforementioned studies, discrepancies remain between evaluation methods. This means that the results and metrics reported by one study cannot always be rigorously compared with all the others, as they must use the same evaluation methods. The evaluation methods featured in these articles are as follows: the evaluation of a validation set (21–26), the evaluation of a test set (27, 28), cross-validation (29–32), and leave-one-subject-out (32, 34, 35).

### Hyperparameters and performance

The benchmark from Table 4 shows that no *a priori* performance ranking of the various algorithms could be made solely based on the type of neural network architecture. Despite our optimized Resnet coming always first, the second best option (where more than three are present) is dependent on the case either based around a CNN architecture (26, 35) or a hybrid-LSTM architecture (32).

This reinforces the claim that hyperparameter search, beyond neural architecture, is key to tune a network specifically to a given dataset and make it reach its best performance. Such hyperparameter studies have systematically been in the focus of top performers (26, 32, 35). They all conducted ablation studies, did trial and errors, or followed best practices from the literature to tune optimally their respective chosen architectures. Cheng et al. found similar results in that regard than our study, that is adding complexity to the network, such as by increasing kernel sizes, leads to higher accuracy and F1-score (26). Lv et al. studied as well as influence of the kernel sizes, the number of layers, and possible fusion modes of outputs of analyzing modules of their hybrid network (32). Jin et al. research mostly focused on designing a custom loss for HAR, the anchor-based loss function that has two loss components balanced by a simple scalar hyperparameter λ that has been investigated and optimized (35).

### Continuous time classification

**Figure 7:**
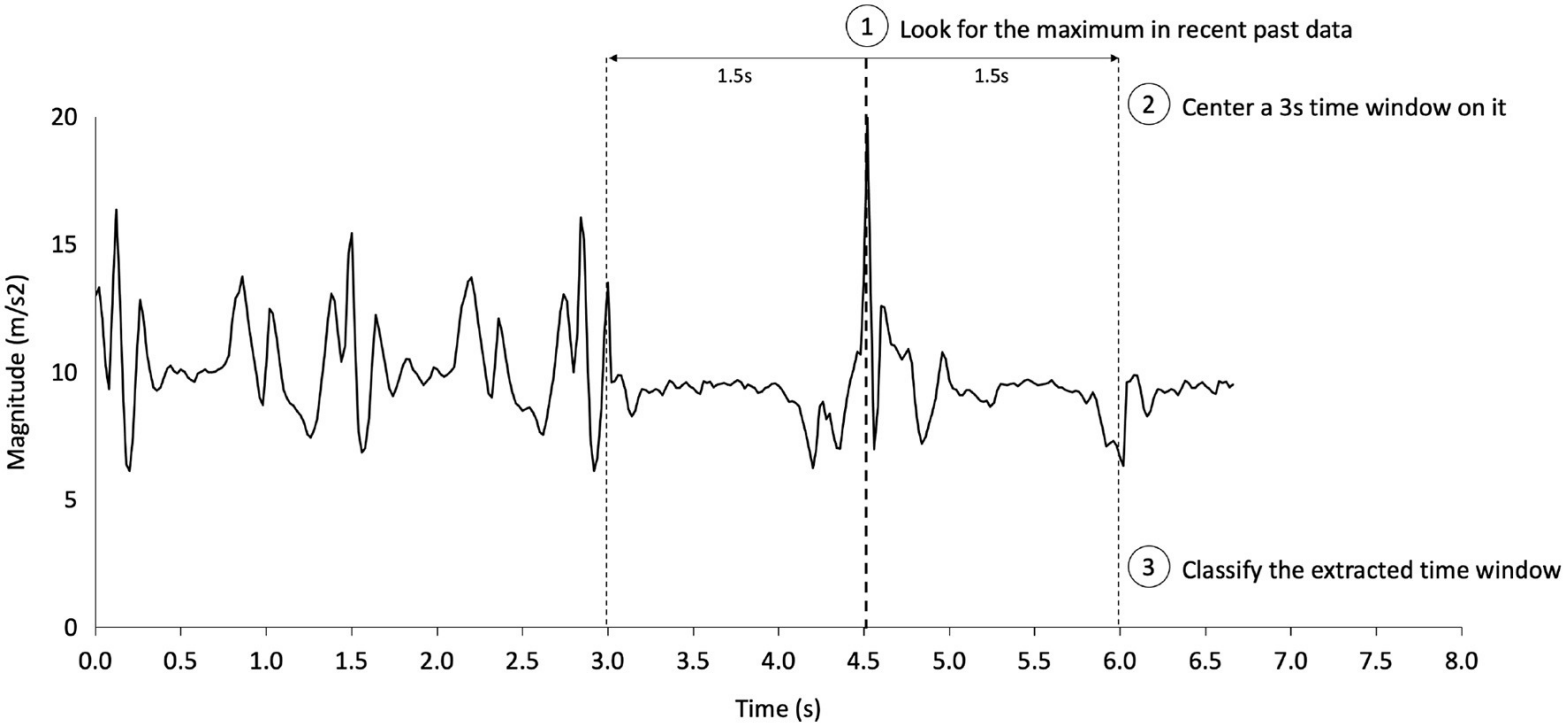
One-step in our proposal for real-time fall detection. This step can then be re-executed at the desired frequency, up to the smartphone's computing power limitations.

Thus far, we have built a classification method based on pre-formatted segments that displays good performance with this framework. We would like to provide perspectives on how a deep learning classifier can be adapted to analyze accelerometer signals in continuous time. We describe the steps of our proposal in Figure 7. This step must be iterated with the desired frequency for real-time analysis. Significantly, the proper implementation of this live analysis could already be tested only with the dataset. All that needs to be done is to concatenate segments of activities beside each other to gain a longer time series featuring multiple activities. For the concatenation to go smoothly and not have abrupt changes, we ensure that distances between the last *x*, *y*, and *z* values of the acceleration of the current time series are near the first ones of the next time series.

### Limitations

Using machine learning for human activity recognition brings up several limitations. First, there is potential for bias in the training data. If the data used to train the model are not representative of the diverse range of activities and individuals, the model may lack robustness and generalizability. A second limitation is a dependence on the quality of the sensors and processing used for recognition. Factors such as noise, sensor placement, and data resolution can all impact the model's accuracy. The third limitation is the computing resources requirement, which corresponds to a machine learning model, to be trained and run effectively. Finally, a fourth limitation revolves around ethical consideration, where privacy, fairness, and transparency should be as carefully considered as performance when deploying such systems running on personal data. In addressing these limitations, it is also important to consider the power consumption implications of using the internal sensors and also processing the data.

## Conclusion

Our research has highlighted the main steps in conceptualizing a deep learning-based human activity recognition classifier. We showed that our optimized Resnet was remarkably fit for this task and that deep learning best practices such as dropout and label smoothing worked well to improve accuracy and the F1-score for fall detection. Our approach raised the state-of-the-art accuracy to 80.09% on the UniMiB-SHAR dataset using the rigorous leave-one-subject-out evaluation method. However, there are still challenges to be addressed on a more global scale, such as collecting data *via* different types of smartphones and diverse populations to reduce bias and increase generalizability.

## Data availability statement

The original contributions presented in the study are included in the article/supplementary material, further inquiries can be directed to the corresponding authors.

## Author contributions

ME designed and led the study. TS, ME, RF, and CM conceived the study. All authors approved the final manuscript.
